# Antibacterial Properties of Gold Nanoparticles in the Modification of Medical Implants: A Systematic Review

**DOI:** 10.3390/pharmaceutics14122654

**Published:** 2022-11-30

**Authors:** Xinxin Zhan, Jianglong Yan, Hao Tang, Dandan Xia, Hong Lin

**Affiliations:** 1 Department of Dental Materials, Peking University School and Hospital of Stomatology & National Center of Stomatology & National Clinical Research Center for Oral Diseases & National Engineering Research Center of Oral Biomaterials and Digital Medical Devices & Beijing Key Laboratory of Digital Stomatology & Research Center of Engineering and Technology for Computerized Dentistry Ministry of Health & NMPA Key Laboratory for Dental Materials, Beijing 100081, China; 2 Pritzker School of Molecular Engineering, The University of Chicago, Chicago, IL 60637, USA

**Keywords:** bacterial infections, medical implants, gold nanoparticles, antibacterial

## Abstract

The widespread occurrence of bacterial infections and their increased resistance to antibiotics has led to the development of antimicrobial coatings for multiple medical implants. Owing to their desirable properties, gold nanoparticles (AuNPs) have been developed as antibacterial agents. This systematic investigation sought to analyze the antibacterial effects of implant material surfaces modified with AuNPs. The data from 27 relevant studies were summed up. The included articles were collected from September 2011 to September 2021. According to the retrieved literature, we found that medical implants modified by AuNPs have good antibacterial effects against gram-positive and gram-negative bacteria, and the antibacterial effects would be improved by near-infrared (NIR) radiation.

## 1. Introduction

Implanted medical devices, such as catheters, cardiovascular stents, orthopedic and dental implants, are commonly used to reduce pain, and improve function and appearance, and can have a significant impact on physical and mental health. However, these devices breach the soft tissue barrier that protects the body from external factors, thereby increasing the risk of bacterial infection, potentially leading to morbidity and mortality [1]. The risk of infection occurs in almost all biomaterial applications, with implant infections being the most common ranging from 0.08% to 40%, depending on the implantation site and time, and the physical health of the patient. Among them, the infection rate related to cardiac devices is the highest [2,3,4].

The mainstay of treatment post-infection is antibiotic therapy. However, as extensively documented, the problem of antibiotic resistance worldwide is increasing, with drug-resistant strains most commonly emerging as a result of antibiotic use in hospitals [5,6]. Therefore, the treatment of device-related infections is increasingly difficult and costly due to long-term antibiotic treatment and the emergence of resistant bacteria. Surgical intervention and removal of devices may be required in cases where antibiotics cannot resolve the infection. However, revision surgeries can carry different levels of risk; one study [7] showed that revision surgery for periprosthetic joint infection was associated with a five-fold increase in mortality compared with revision surgery for aseptic failure.

In addition to antibiotics use, many methods have emerged to tackle the problem of bacterial infections associated with medical implants; for example, modifications to the basic composition of the implant or the surface of the implant, such as antimicrobial peptides (AMPs) [8,9], quaternary ammonium compounds, cationic materials, metal ions [10], nanoparticles, and photodynamic therapy, have been developed to prevent infection. The unique amino acid sequences of AMPs enable them to insert into and decompose on the surface of bacteria, thus, killing drug-resistant bacteria [11]. However, AMPs also have several disadvantages, including poor biodistribution, frequent toxicity, hemolytic activity, rapid degradation, and other side effects [12,13,14]. A recurring reason for the failure of AMPs to reach the market is that they do not show better activity than currently available antibiotics for specific indications [15]. Many quaternary ammonium compounds not only have antibacterial effects but also have antifungal, antiviral, and antimatrix protease capabilities. A promising strategy for preparing antimicrobial biomaterials is to incorporate quaternary ammonium moieties into polymers; however, these technological developments can also pose challenges in terms of toxicology and antimicrobial resistance [16]. Positively charged long-chain polymers show potent bactericidal activity by penetrating bacterial membranes [17] and are widely used in drug delivery, gene delivery, and tissue engineering [18]. When designing cationic polymers, it is important to consider methods of overcoming subcellular barriers, including in vivo escape and nuclear transfer. The success of cationic polymer is hindered by the non-degradability and toxicity of some formulations [19]. Inorganic nanoparticles with a large specific surface area, size control, flexible surface functionality, relatively good biocompatibility, and additional properties, such as photocatalysis, photothermal effects, and reactive oxygen species stimulation, show good antibacterial potential [20,21]. Nanoparticles are less likely to promote bacterial resistance than antibiotics, because nanoparticles can contact directly with bacterial cell walls [22].

Among inorganic nanomaterials, the research on AuNPs is increasing, and their advantages as antibacterial agents include: highly biosafe; can be designed to regulate gold nanomaterial excretion/metabolism; different molecules can be used to modify the surface; can enhance antibacterial effects by manipulating size, shape, and surface properties; and can seldom induce bacterial resistance [23]. Moreover, drug-coupled AuNPs showed higher and longer antibacterial effects [24,25]. In this review, we investigated the applications of medical implants and summarized the research on the antibacterial properties of AuNPs. We focused primarily on the possible modification method on medical implants, the antibacterial effects of implant materials modified by AuNPs, the main pathogenic bacteria which they against, and their antibacterial mechanism.

## 2. Methods

**Figure 1:**
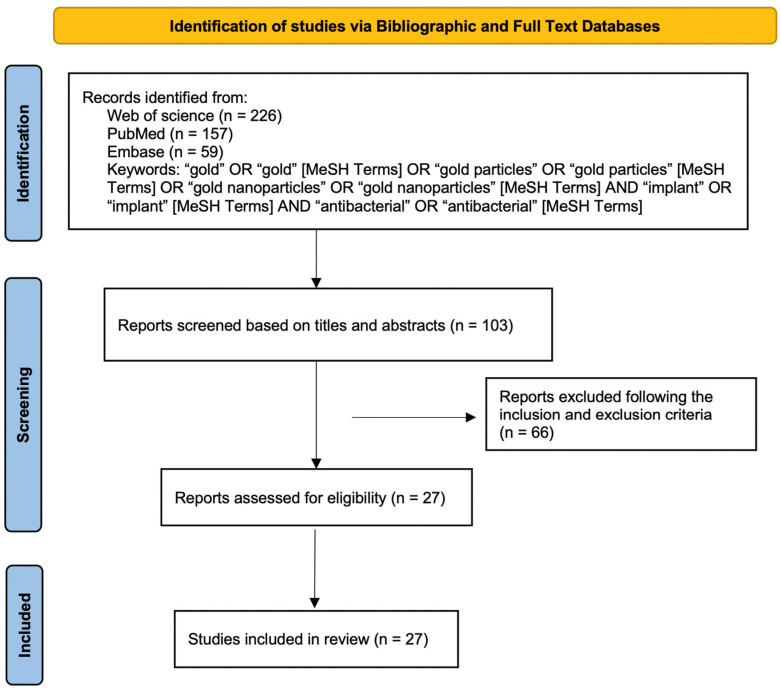
PRISMA flow diagram.

This review was conducted in accordance with the PRISMA systematic review statement guidelines [26]. Figure 1 shows a flowchart describing the selection, identification, and screening methodology for the studies of interest in this review article.

### 2.1. Electronic Resources/Bibliographic and Full Text Databases

For the research strategy, the following databases were used: (i) PubMed; (ii) Web of Science; (iii) Embase. Keywords were used as follows: “gold” OR “gold” (MeSH Terms) OR “gold particles” OR “gold particles” (MeSH Terms) OR “gold nanoparticles” OR “gold nanoparticles” (MeSH Terms) AND “implant” OR “implant” (MeSH Terms) AND “antibacterial” OR “antibacterial” (MeSH Terms).

### 2.2. Study Selection

The inclusion criteria were as follows: (1) the research materials were primarily used for medical implants, (2) AuNPs were applied to implants in different ways, such as by changing the basic composition, surface modification, and surface modification in combination with other components, (3) AuNPs were used for antibacterial purposes. The exclusion criteria were as follows: (1) the study was a duplicate, (2) the study materials were not applied to medical implants, (3) AuNPs were not used, (4) papers were reviews or systematic reviews.

The first step in the research selection strategy was to identify relevant papers, we selected 226 documents from Web of Science, 157 from PubMed, and 59 from Embase. All retrieved articles were selected based on their titles and abstracts, and articles that were not relevant to the subject area were excluded, leaving 103. Potentially suitable articles were evaluated according to the inclusion and exclusion criteria. For more detailed screening, the full texts of the remaining articles were evaluated. Finally, 27 articles that met the eligibility criteria were selected and discussed.

### 2.3. Data Extraction and Synthesis

Data relating to the characteristics of the included studies were extracted by one reviewer and verified by another reviewer (Table 1).

## 3. Results

### 3.1. Implant Materials for Medical Applications Studied in Selected Articles

**Figure 2:**
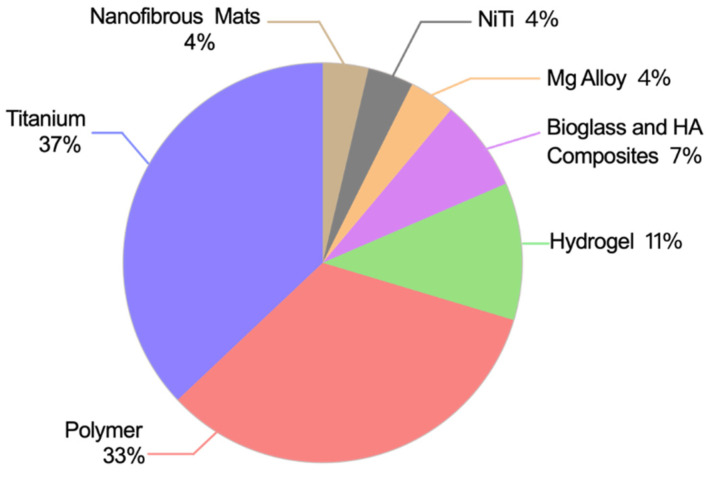
The varieties and numbers of substrate materials in publications.

The substrate materials modified in the 27 articles mainly included metals, polymers, and bioactive materials (Figure 2). There were ten articles on the surface modification of pure titanium (Ti), including eight studies on titania nanotube (TNT) modification [30,31,32,33,34,35,36] and two studies on titanium surface modification [37,38]. Two studies [51,52] presented AuNPs that were used to improve the antibacterial effects of magnesium (Mg) alloys and nickel titanium. Three hydrogel composites doped with AuNPs have been investigated [27,28,29]. In nine of the studies [39,40,41,42,43,44,45,46,48], AuNPs were used to modify polymers, including polymethyl methacrylate (PMMA)-based bone cement, polyurethane (PU), polypropylene, polydimethylsiloxane (PDMS), and polyvinyl alcohol (PVA). There were two studies [49,50] regarding bioglasses and hydroxyapatite. There was one article on AuNP modifications for nanofibrous mats [47].

### 3.2. The Modification Methods of AuNPs to Implant Materials

**Figure 3:**
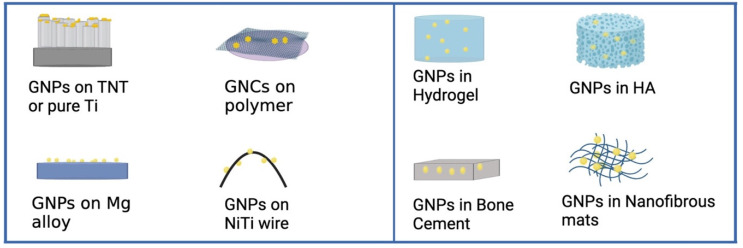
The locations of gold nanoparticles after implant material modification.

The modification methods of gold nanoparticles to implant materials mainly include immersion, sputtering, self-assembly, mixing. After implant materials modification, gold nanoparticles can be present on the surface of the implant materials or inside the implant materials. The locations of gold nanoparticles are illustrated in Figure 3. The AuNPs or gold nanorods (AuNRs) can be directly coated onto titanium dioxide nanotubes, pure titanium, hydroxyapatite particles, or polymers using simple immersion, sputtering methods or self-assembly methods. Furthermore, titanium dioxide nanotubes and PDMS can be soaked in a chloroauric acid solution, and the AuNPs obtained by reduction were added to the surface of the material under UV light. On the other hand, the gold nanoparticles can be mixed into implant materials. Different amounts of AuNPs can be directly mixed with polymers to form composites. Besides, chloroauric acid solution can be in situ reduced by chitosan to gold nanoparticles and mixed into form hydrogel composites. It is worth noting that in the coating preparation process, in order to improve the binding ability and therapeutic effect of the coating, the AuNPs or the surface of the substrate materials may need to be pretreated by chemical reaction, such as Polyethylene glycolation (PEGylation).

### 3.3. Application Sites and Antibacterial Research of Medical Implants in Selected Articles

**Figure 4:**
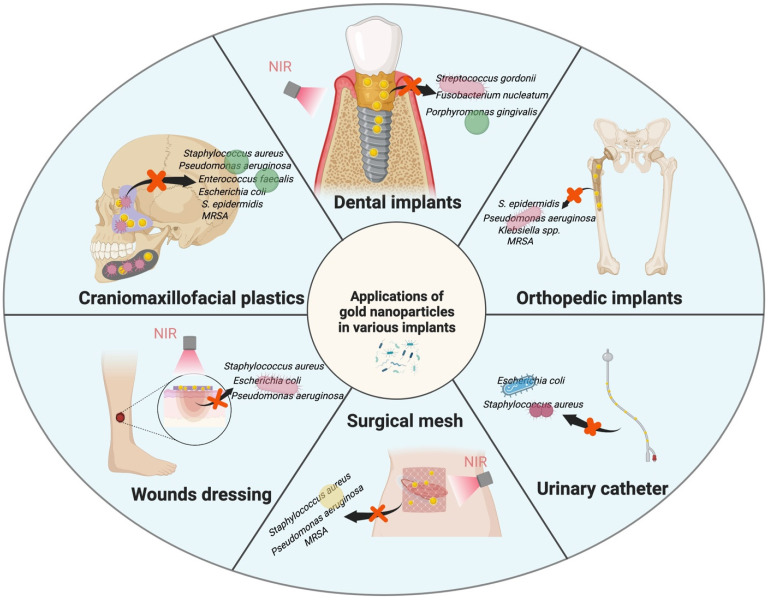
Applications of gold nanoparticles in various implants.

Most modified medical implants are used for bone defect treatments [31,33,34,35,36,37,38,43,49], dental implants [30,32], drug delivery [25,50], prosthetic joints [29,41,52], and medical devices [44,51]. Modified hydrogels are commonly used in wound dressings [27,28]. Polymeric materials are used in catheters [40,48], hernia repairs [39,45], and menisci [42]. The key application sites presented across the 27 papers analyzed are shown in Figure 4. Gram-positive bacteria, including *Staphylococcus aureus* and *S. epidermidis*, and gram-negative bacteria, such as *Escherichia coli* and *Pseudomonas aeruginosa*, are used in most antibacterial assays. According to the different infection sites, some researchers have selected targeted bacteria (Figure 4), such as *Streptococcus mutans* [25,35], *Porphyromonas gingivalis* [30,32], *Klebsiella* spp. [42], and *Enterococcus faecalis* [53]. The emergence of drug-resistant bacteria [38,41,44], such as methicillin-resistant Staphylococcus, is an important driving force for researchers seeking alternative antibacterial methods to antibiotics.

With increasing concentration, the antibacterial capacity increased. However, as the dose increases, gold nanoparticles may adversely affect the properties of the substrate materials. By harnessing the photothermal effect of gold nanoparticles, drug release, such as Vancomycin [36] or Tetracycline [25,35], could be controlled to synergistically improve antimicrobial effects. Moreover, when the temperature exceeds 50 °C, bacterial proteins denature and bacteria can be killed.

### 3.4. The Antibacterial Mechanism of Gold Nanoparticles

**Figure 5:**
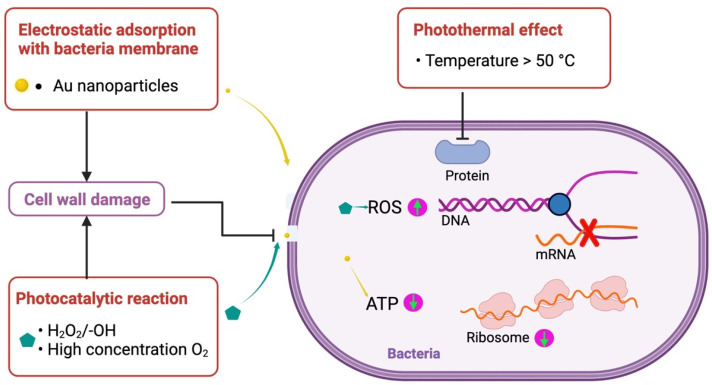
Schematic illustration of antibacterial mechanism of gold nanoparticles.

The implant materials modified with gold nanoparticles exhibit good antimicrobial effects, largely thanks to gold nanoparticles. The antimicrobial mechanism of gold nanoparticles is shown in Figure 5. Gold nanoparticles electrostatically adsorb to the bacterial membrane and can interact strongly with lysine present on the bacterial membrane of gram-positive bacteria [28,50], where irreparable pores appear in the bacterial membrane and cause bacterial death. Moreover, after entering into bacteria, gold nanoparticles reduced adenosine triphosphate (ATP) levels and led to decreased metabolism [54]. Gold nanoparticles promote the photocatalytic activity of oxides, such as titanium dioxide [32] and zinc oxide [40], and produce peroxides, hydroxyl groups, and high concentrations of oxygen [30], which generate excess ROS [31] and cause bacterial collapse. Under near-infrared light (NIR), gold nanoparticles have excellent photothermal effects. When temperatures are above 50 °C, the bacteria are ablated due to protein deformation.

### 3.5. Biocompatibility of Implant Materials Modified by Au Nanoparticles

Of the included articles, most also investigated the biocompatibility of modified implant materials. Cytotoxic and inflammatory responses have been previously described; however, none of the modified surfaces exhibited significant cytotoxicity in vitro. Most AuNPs were approximately 20 nm in size. The addition of 5% gold content to the surface of TNT showed the best anti-inflammatory effect [30], promoted initial adhesion, and enhanced the spreading and proliferation of rat bone marrow mesenchymal stem cells (rBMSCs) [31], evenly stimulating the ALP activity of rBMSCs [31,43]. However, one study showed that chitosan hydrogels with AuNPs led to a higher thickness of the fibrous tissue capsule (approximately 80–100 μm).

## 4. Discussion

### 4.1. Antibacterial Importance of Medical Implants

Biofilm formation in biomedical implants and devices is a serious issue. The negative effects of biofilms have been well studied and documented in various biomedical settings [55]. The long-term use of biomaterials in the body is threatened by bacterial adhesion and proliferation on the implant surface, leading to biofilm formation in some cases; this can lead to local infection and even implant failure, which in the worst cases leads to patient death [56,57].

Implant-related infections trigger local tissue responses, leading to acute and chronic inflammation, foreign body reactions, granulation tissue formation, and fibrous encapsulation. These events may ultimately drive microbial colonization and implant infection. Implant infections are therefore characterized by complex interactions between biomaterials and the host, especially the host immune response. Biofilm formation is often responsible for the development of nosocomial infections. Once a biofilm has formed, it protects the adherent bacteria from host defense systems and fungicides through a variety of mechanisms. Biofilms can form on virtually any material present in the operating room, and preventing their formation is fundamental to patient survival [58].

The systemic application of antibiotics is currently the primary treatment modality for infection, but the overuse of antibiotics has led to the microevolution of resistant bacteria. Some bacterial strains are now showing resistance to all commonly used antibiotics; for example, methicillin-resistant golden yellow *Staphylococcus* has developed resistance not only to methicillin but also to macrolides, aminoglycosides, lincosamides, and chloramphenicol [59]. By making the implant surface antimicrobial, the adverse effects of the systemic use of antibiotics and the delivery of large doses to implant sites can be avoided [60]. The key to limiting the spread of infection after the installation of medical implants is to prevent bacterial colonization of biomaterial surfaces. The properties of the material determine the general mechanical behavior, and the biological activity is closely related to the surface properties [57]; therefore, the improvement of surface functionalization is important in improving the biological functions of medical implants, such as their antibacterial properties.

### 4.2. The Characteristics of AuNPs Determine the Antibacterial Effects

The advantages of AuNPs as antibacterial agents include the following: (1) Gold nanomaterials can be endowed with high biosafety because gold itself is chemically inert, and the absorption/metabolism of gold nanomaterials can be regulated by the material design. (2) The antibacterial effects of AuNPs can be maximized by the chemical manipulation of properties, such as size, shape, and surface, by modifying the surface with different molecules. (3) Gold nanomaterials induce bacterial resistance less frequently than standard antibiotics [23]. (4) Additionally, gold nanoparticles can be functionalized by natural antioxidant, biological ligands, various organic molecules, and dendrimer. The functionalized gold nanomaterials have several advantages in surface charge, size, bacterial receptor targeting, biocompatibility, and effective internalization [61]. In our systematic review, we summarized the antibacterial effects of medical implants modified with AuNPs, the current feasible modification methods, the types of modified implant materials, the antibacterial effects on microorganisms, and the potential limitations of this technology.

Titanium and titanium alloys are important in orthopedic implants. Titanium dioxide nanotubes can be formed on the surface of titanium using anodizing treatment. Titanium dioxide nanotubes (TNT) are similar to natural bone matrices because of their nanoscale structure and the use of drugs. Pristine TiO_2_ does not possess antibacterial capabilities unless catalyzed by ultraviolet (UV) light, which cannot penetrate tissue to reach implants in vivo [33]. Light in the near-infrared region (650–900 nm) has a lower rate of optical adsorption by body components, such as water, and would therefore allow deep tissue penetration without significant damage [36]. Infrared light is more efficient than visible light because it has better skin penetration [35]. When TiO_2_-NTs are decorated with AuNPs, their photothermal effect of AuNPs enables them to achieve enhanced photocatalysis [30,32]. There are also studies showing that without near-infrared laser excitation, titanium dioxide nanotubes modified by AuNPs have good antibacterial effects on *Streptococcus gordonii, Porphyromonas gingivalis,* and *Fusobacterium nucleatum*, and the in vivo results showed a reduced inflammatory response. Further, research has shown that the surface of AuNP-modified TiO_2_ nanotubes exhibits a superior antibacterial effect on *E. coli* under NIR laser irradiation.

AuNPs with special designs, such as nanoshells and nanorods, strongly absorb light in the near-infrared (NIR) spectrum and convert it to heat. Research [62] elaborated that gold nanoparticles have the ability to localize light to sub-wavelength regions. Additionally, under light, gold nanoparticles produce a field-enhancing effect. In addition, the optical character of nanoparticles is affected by its size, shape, and dielectric environment [63]. The peak magnitude of the field for the 30 nm particles is larger than that of 60 nm particles. The particle shape strongly affects the plasmon resonance, which shifts to red as the particles becomes more oblate. This photothermal effect is due to the surface plasmon resonance (SPR) effect of gold nanoparticles under the action of near-infrared [64].

AuNPs are mainly in round, rod, nanostar, or nano-flower formations. Different shapes have different antibacterial effects, and it has been suggested that nanostars have the highest antibacterial activity [65]. However, with regards to implant material modifications, the particle shape was not shown to have a significant antibacterial effect, but this might be due to the fact that these studies did not only consider AuNPs as the factors that act on microorganisms. In a previous study [65], the results showed that the antibacterial effect depended on the number of AuNPs. A small increase in the number of AuNPs enhanced the anti-biofilm activity of the nanocomposite, inhibiting 99.99% of *Klebsiella* spp. and *Staphylococcus epidermidis* [42]. However, with regards to TiO_2_ nanotubes, we found that AuNP content of less than 10 wt% could spread evenly on the titanium surface, and when the content was too high, the nanoparticles tended to aggregate [33]. For AuNPs mixed into implant materials, the dose affects the basic mechanical properties of the substrate material [41] and the increase in dose does not affect cell compatibility.

From the included studies, we found variations in shape, size, and the content of AuNPs. We found that shape of AuNPs was not the main factor influencing the antibacterial effect. In most studies, concentrations below 10 wt% showed antimicrobial effects, but this relied on synergistic effects with other components. AuNPs can be functionalized by specific factors to achieve targeted and laser thermal ablation to kill bacterial [66]. In addition, the antibacterial effect of functionalized AuNPs is related to particle aggregation [67,68], and the tunability of optical response and catalytic activity is achieved through the controllable aggregation of functionalized AuNPs. The antibacterial effect of AgNPs is well known, and the addition of AuNPs enables the uniform dispersion of silver/AuNPs and a synergistic effect [51]. AuNPs have also shown a synergistic effect with antibiotics and cations against the gram-positive bacteria *Staphylococcus aureus* and gram-negative bacteria *Escherichia coli* [27,52], as well as improving drug release [69].

### 4.3. Influence of NIR on Antibacterial Effects of AuNPs

In the 27 included articles, we found that studies that presented thermal or photocatalytic effects by using NIR on AuNPs. Most AuNP therapeutic applications are based on their ability to generate tunable heat upon exposure to NIR radiation, which is helpful in both NIR-responsive cargo delivery and photothermal/photodynamic therapies [70,71]. AuNPs exhibited remarkable absorption in the NIR region.

Protein activity is affected by protein unfolding and aggregation in the temperature range 43–45 °C, but in the case of NIR radiation, the temperature does not induce these modifications at the cellular level [72]. It has been reported that, above 50 °C, the enzymes, proteins, and lipids in bacteria become denatured and metabolism is disordered, eventually resulting in bacterial death [73].

NIR light has been shown to travel at least 10 cm through breast tissue, and 4 cm of skull/brain tissue or deep muscle using microwatt laser sources [74]. This indicates that NIR can effectively excite medical devices implanted in the body, without resulting in damage to the patient.

### 4.4. Antimicrobial Mechanism

The antibacterial effect is related to the morphology [75] and bacterial membrane [76] of bacteria, and the different forms of bacteria make the contact area between bacteria and the sample surface different. The composition of the cell wall of gram-negative bacteria is different from that of gram-positive bacteria; the peptidoglycan layer in the cytoderm of gram-positive cells is much thicker than that of gram-negative cells, and it is therefore batter suited to resisting influx of metal ions [77]. Lysine is the most abundant amino acid in gram-positive bacteria [78] and is characterized by a –CH_2_CH_2_CH_2_NH_2_ group that strongly interacts with AuNPs; this may be detrimental to gram-positive cells. After contact with bacteria, AuNPs pass through the outer membrane and peptidoglycan layers of bacteria, generating reactive oxygen species (ROSs) [79], which can interrupt the extracellular electron transfer pathway that contacts bacteria, ultimately affecting bacterial growth.

On the other hand, it is related to the surface properties of the materials [75]. The physical action of nanopillars can cause bacterial deformation, and the PEGylated hydrophobic surface reduces bacterial adhesion [45]. In the example of TNT-Au modification [31,32,33], the addition of AuNPs can expand the light absorption range, improve photocatalytic activity, generate more hydroxyl groups and superoxides, and increase the biosafety of the material. Nanoparticles have a larger surface area for interaction to enhance the bactericidal effect than larger size particles, and with the addition of AuNPs, they are able to bind to the functional groups of proteins, resulting in protein inactivation and denaturation. The antibacterial mechanism of nanoparticles is dependent on their sizes [80]. Because of their small size [81], AuNPs can easily penetrate the bacterial wall, and the DNA molecules condense and lose their ability to replicate, leading to cell death. In addition, the penetration of bacterial cell walls by AuNPs causes enzyme inactivation, the production of hydrogen peroxide, and, ultimately, bacterial cell death [82].

AuNPs produce photothermal effects under NIR irradiation, and the increase in temperature produces thermal ablations on bacteria, especially Gram-negative bacteria. A study [83] showed that high temperatures can affect *Escherichia coli* outer membrane protein folding. An increase in temperature leads to protein denaturation [84] and the loss of enzyme activity, inhibiting bacterial growth [72]. The photothermal effect of gold also assists in the release of antibiotics added to the surface of the material [35,36], increasing antibacterial efficacy and long-term antibacterial effects [80].

### 4.5. Biocompatibility of Implant Materials Modified by AuNPs

Biomaterials come into contact with cell surfaces, tissues, organs, and blood components. The toxicity assessment of implant materials is a principal issue for potential medical applications [85]. Gold nanoparticles have good biocompatibility at the appropriate concentration and size, and excessive concentrations will cause toxicity. Some literature illustrated that gold nanoparticles with reasonable size and concentration have good cytocompatibility [33,37,42]. The results of cytocompatibility studies showed that AuNPs (of approximately 20 nm) did not cause HDF-f cell death at a maximum concentration of 300 μM [86]. The 20 nm AuNPs exhibited the lowest uptake by reticuloendothelial cells and the slowest clearance from the body [87]. As the size of the AuNPs increased, the permeability and diffusion coefficients decreased [88].The results of an in vivo experiment showed that AuNPs of 3, 5, 50, and 100 nm did not show harmful effects, whereas AuNPs ranging from 8 to 37 nm induced severe sickness in mice at a dose of 8 mg/kg per week [89]. Other studies showed that 10 nm nanoparticles [90] and 15 nm nanoparticles [91] both showed the most widespread organ distribution.

In addition, in antimicrobial strategies for bone defect restoration and dental implants, AuNPs exhibit good biological activity, such as in the promotion of fibroblast adhesion, proliferation, and migration [32]. Moreover, AuNPs can stimulate osteogenic differentiation of bone marrow mesenchymal stem cells (BMSCs) [31,43] and promote the bone-forming effect of implant materials [92]. Osteogenesis is significantly promoted when combined with bioactive materials [43]. Furthermore, gold nanoparticles exhibit anti-inflammatory effects [30,38,45]. In wound-dressing antibacterial applications, AuNPs exhibit good collagen fiber regeneration to promote wound healing [28].

## 5. Conclusions

In conclusion, gold nanoparticles could modify implant materials by sputtering, immersion, self-assembly, mix, etc. Gold nanoparticles have good effective against bacteria, and this effect can be enhanced by an NIR laser. The antimicrobial effect of gold nanoparticles was dose-dependent. Considering the surface properties of the material, the gold nanoparticle content is preferably not more than 10%; approximately 5% is the most favorable for the inflammatory response. Gold nanoparticles with a size of approximately 20 nm are the most conducive to biocompatibility. Based on the good biological properties of gold nanoparticles, they have broad prospects in the field of antibacterial researches of medical implant materials.

## Figures and Tables

**Table 1:** The antibacterial effects and other effects of implant materials modified by various gold nanoparticles.

Materials	Application	Au Size	Au Concentration	External Stimulation	Antibacterial Effect	Other Effects	Ref.
nAu-Hydrogel	Wound dressings	<20 nm	1 mM HAuCl_4_ solution	-	Considerable antimicrobial activity for *S. aureus* and *P. aeruginosa*.	The thickness of capsule is higher (∼80–100 μm) in the case of AC-nAu.	[27]
Au@PDA nanocomposite Hydrogel	Wound dressings	Length, 40 nm; diameter, 10 nm	42 mg/g of Au@PDA	Near-infrared (NIR), 2 W cm^−2^ 808 nm, 5 min	98% killing efficiency against *S. aureus* and *E. coli*.	Promote wound healing of infected full-skin defect.	[28]
AuNRs_mPEG Hydrogel	Prosthetic joint infection replacement	Length, 110 nm; diameter, 30 nm	300 ppm	NIR, 1 W cm^−2^ 808 nm, 20 min	20 min of PTT following the initial 2 h D-AA treatment is sufficient to remove *S. aureus* biofilm.	-	[29]
TNTs/AuNPs	Dental implants	20 nm	-	-	The average antibacterial efficiency of TNT-Au2 sample is 97.34% (*P. gingivalis*) and 92.13% (F. nucleatum).	High anti-inflammatory efficiency.	[30]
TNTs/AuNPs	Bone defect implants	-	5.3 at. % 8.5 at. %	-	An antibacterial effect towards both *S. aureus* and *E. coli*.	Promote initial adhesion, the spreading and proliferation of rBMSCs.	[31]
TNTs/AuNPs	Dental implants	-	6.52 at. % 2.01 at. % 1.59 at. %	-	Enhanced antibacterial activity with Au content increasing, >99% inhibition against multispecies biofilm.	Promote fibroblast adhesion, proliferation, and migration.	[32]
TNTs/AuNPs	Bone defect implants	10 nm and 20 nm	15–40%	-	Long-term antibacterial characteristics against *S. aureus*.	Good cytocompatibility.	[33]
TNTs/Ag and Au NPs	Bone defect implants	5–20 nm	0.30%	-	Ag and Au have a synergistic effect on *E. coli*.	-	[34]
TNTs/AuNRs	Bone defect implants	Diameter, 10 nm; aspect ratio, 3.83	<12 wt %	NIR, 200 mW, 830 nm, 30 s	Tetracycline was released effectively by NIR, showed the annihilation effect of Streptococcus mutans.	-	[35]
TNTs/AuNRs	Bone defect implants	Diameter 10 nm	2.8 nM	NIR, 1 W cm^−2^ 850 nm, 30 min	Vancomycin released from the coating induced by NIR, resulting in a clear inhibition zone to Staphylococcus epidermidis.	-	[36]
TNTs/AuNRs	Bone defect implants	Diameter, 35 nm; length, 100 nm	5.03 wt %	NIR, 200 mW, 830 nm, 30 min	The zone index of *S. mutans* grown with 2 wt% TC/PCL-coated GNRs-TNT following NIR laser irradiation for 1 min (16.25 ± 1.39 cm) was significantly higher.	-	[25]
Ti-AuNRs	Bone defect implants	Diameter, 11 nm; length, 50 nm	0.02 M, 0.12 mL HAuCl_4_ solution	NIR, 0.5 W cm^−2^, 808 nm, 20 min	The antibacterial activity of Ti-GNR-NIR group is highest in *E. coli*, *P. aeruginosa*, *S. aureus*, and *S. epidermidis*.	An ignorable toxicity to MC3T3-E1 cells.	[37]
Network Films-AuNCs	Bone defect implants	-	3 mM HAuCl_4_ solution	-	Disrupt the MRSA and ESBL *E. coli* membrane.	No obvious tissue defect.	[38]
Surgical mesh-AuNRs	Hernia repair surgical mesh	-	250 GNRs/μm^2^	NIR, 0.435 W cm^−2^, 810 nm, 30 s	Alter the integrity of biofilm.	-	[39]
PDMS- ZnO/Au	Urinary catheters	-	10 mg/mL HAuCl_4_ solution	Visible light	A killing rate of 65.5% in the dark and >99.9% under visible light irradiation and obstruct the attachment of *E. coli* bacteria.	-	[40]
Bone cement-AuNPs	Total knee arthroplasty and total hip replacement	10–20 nm	0.25 wt % 0.5 wt %, 1 wt %	-	Live bacteria reduced up to 54% and 56% for MRSA and Pseudomonas, respectively, on bone cement samples obtained by adding 1% by weight of AuNPs.	0.25 wt% AuNPs improved the punching performances, without altering the compressive properties of bone cement.	[41]
PU-AuNPs	Menisci	-	0.16 wt % 0.32 wt % 0.64 wt %	-	0.64 wt % inhibiting 99.99% of *Klebsiella* spp. and *S. epidermidis*.	Do not exhibit toxic effects on fibroblast cells.	[42]
CS/PVA/GO/HAP/Au nanocomposite	Bone tissue engineering scaffolds	-	-	-	An increase of 3–7 mm zone of inhibition was seen in Cs/PVA/GO/HAP/Au film.	High hemocompatibility, not toxic, active differentiation of mMSCs.	[43]
PDMS-TA-PEG-Au	Biomedical devices	40–70 nm	-	NIR, 1.5 W cm^−2^, 808 nm, 10 min	Resists MRSA bacterial adhesion, kills *S. aureus* and *E. coli* bacteria in vitro and in vivo.	Biocompatible with low cytotoxicity.	[44]
PU-Au-PEG	Hernia repair	Diameter, 10 nm; length, 40 nm	10.84 wt % 12.52 wt %	NIR, 1.2 W cm^−2^, 808 nm, 10 min	Kills *P. aeruginosa* and *S. aureus*, inhibits biofilm formation, eliminates infection of the hernia.	Good biocompatibility.	[45]
PVA-AuNS	Wound dressings	-	9 × 10^−3^ M	NIR, 0.3 W cm^−2^, 800 nm, 30 min	*S. aureus* and *E. coli* film can be killed if laser treatment as short as 30 min is applied.	-	[46]
Nanofibrous mats-AuNPs	Tissue engineering scaffolds	20 nm	-	-	Better antibacterial effect against *S. aureus* than *E. coli*.	-	[47]
PDMS-AuNPs-GO-NH2	Urinary catheters	1.4 nm	-	-	The bactericidal efficiency of Au-GO-NH2 modified PDMS > 99.99%.	-	[48]
HAp/AuNPs	Bone defect implants	-	-	-	Strong antimicrobial activity (cell mortality > 95%) against *E. coli* and *S. aureus*.	Approximately 90% viability at MIC strength of the nanoparticles.	[49]
58S bioglasses/AuNPs	Drug delivery	<10 nm	0.1 wt.% 1 wt.%	-	Enhances this effect on the *S. aureus* but not on the *E. coli*, the antibacterial effect is dose-dependent.	-	[50]
Mg alloy-AuNPs/PD	Bone fixation plates, screws, wires, pins, and stents	150 nm	-	-	The coating with Ag and Au NPs showed the highest antibacterial effects.	Improved the corrosion resistance of the bare alloy.	[51]
NiTi-AuNPs/CS	Orthopedics and dentistry	-	-	-	AuNPs/CS/NiTi shows highest growth inhibition for *S. aureus*.	High corrosion resistance at all pHs.	[52]

## Data Availability

Not applicable.
